# Internet Influence of Assisted Reproduction Technology Centers in China: Qualitative Study Based on WeChat Official Accounts

**DOI:** 10.2196/17997

**Published:** 2020-06-10

**Authors:** Fang Shao, Zhiqiang He, Zheng Zhu, Xiang Wang, Jianping Zhang, Jinhua Shan, Jiajia Pan, Hui Wang

**Affiliations:** 1 Department of Biostatistics, School of Public Health Nanjing Medical University Nanjing China; 2 School of Pediatrics Nanjing Medical University Nanjing China; 3 School of Nursing Nanjing Medical University Nanjing China; 4 School of Public Health Nanjing Medical University Nanjing China; 5 Department of Histology and Embryology, School of Basic Medical Sciences Nanjing Medical University Nanjing China; 6 State Key Laboratory of Reproductive Medicine Nanjing Medical University Nanjing China

**Keywords:** ART center, WeChat official account, Internet influence

## Abstract

**Background:**

The prevalence of infertility in China is high, but the advent of assisted reproduction technology (ART) has greatly eased this situation. Social media, such as WeChat official accounts, have become the preferred tool for ART centers to communicate with patients, but their attention and operational status differ, and the Internet influence of WeChat official accounts is insufficient. In addition, questions about whether Internet influence is consistent with academic influence and whether the Internet can influence patients’ choice of medical treatment to a certain extent have not been explored.

**Objective:**

This study aimed to examine the operational status and Internet influence of WeChat official accounts for ART centers and to explore the degree of Internet influence on patients’ choices of medical treatment.

**Methods:**

We collected information from the WeChat official accounts for ART centers approved by the National Health Commission of the People’s Republic of China and used the technique for order of preference by similarity to ideal solution to build an Internet influence model of the ART centers and obtained a Ranking of Internet Influence on Reproductive Centers (RIIRC) for each center.

**Results:**

We found there were 451 ART centers throughout the country by the end of 2016 and 498 by the end of 2018. The number of medical institutions is quite large, but their distribution is uneven, and their level of medical technical ability is very different. Analysis of the text data of posts of WeChat official accounts showed the ART centers have insufficient awareness of network exposure and publicity, and the RIIRC of some medical institutions was inconsistent with their medical level and academic status.

**Conclusions:**

ART institutions have varying degrees of emphasis and use of WeChat official accounts in China. They fail to realize that the Internet influence of WeChat may bring them potential patient resources and that Internet influence may affect the future market structure of ART and may also potentially affect academic rankings.

## Introduction

### Severe Infertility and the Increased Requirements of Assisted Reproduction Technology Service

The prevalence of infertility in China, which has increased from 6.7% in 1988 to 15% to 20% now [1,2], has been influenced by a number of factors, including marital status, educational level, delayed age of child-bearing, the two-child policy, repeatedly induced abortions, poor lifestyle, and environmental pollution. Infertility has brought a heavy burden on countless families and has had a number of important effects on both personal and public health [3-6], including damage to social reputation [7], increased psychological pressure [8], long-term infertility [9,10], and constraints on economic development [11].Infertility is not only a physical condition but also a complicated sociological problem in China. However, it is possible to achieve pregnancy through assisted reproductive technology (ART) in cases that cannot be successfully addressed by conventional drugs or surgery.

The National Health Commission of the People’s Republic of China (NHCPRC) approved the implementation of human ART licenses in medical institutions more than 30 years ago. However, it was not until 2007 that the State Council delegated approval authority to the provincial health commissions. Before then, there were only 95 ART centers in China [12], and the number of these centers was initially limited, and their growth was slow because of the technical barriers to ART. Since the decentralization of approval authority and the development of the second and third generations of ART, these centers grew rapidly, and by the end of 2018, there were 498 such centers in China [13]. However, this rapid growth has led to uneven development in terms of the geographical distribution and the level of medical technology.

### Competition in Assisted Reproduction Technology Market Approaches into the Qualitative Stage

In 2015, the NHCPRC proposed there should be *one institution for every 3 million people* to plan the development of ART centers [14]. The total number of ART centers in China under this policy was set at about 550, and the number of ART centers is approaching this limit, which is expected to result in a new stage of qualitative growth rather than quantitative growth. As it is extremely unlikely that there will be new public medical institutions, private capital investment is making competition in the ART market increasingly fierce. Many private investors have entered into the 100 billion market, such as Chengdu Xi’nan Gynecology Hospital and China IVF Medical Group Medical Group. Therefore, the focus of the ART centers in this next stage should be not only to upgrade their medical technology but to expand their public presence to attract the attention of potential patients.

### Ignorance of Internet Images Influencing Patients’ Choice by Assisted Reproduction Technology Centers

Recent statistics indicate that the number of mobile internet users in China has reached 847 million, of which 48.3% are between 20 and 40 years of age [15]—the so-called generation of “Cyberspace natives.” They are accustomed to searching for information on the internet and forming their own opinions, which guides their decision in selecting medical institutions, but they may not pay much attention to the academic standing of medical institutions. This age group is also the main population that is interested in pregnancy preparation, and many of them are currently undergoing ART.

In contrast, most hospitals, including their ART centers, have ignored the impact of the internet, focusing instead on feedback from patients. In the highly competitive medical market, the technology and equipment of ART centers are increasingly easy to imitate and replace, and it is difficult for their hospitals to create their own unique identity. As the costs of infertility diagnosis and treatment are not covered by essential national medicare and the role of Cyberspace natives, internet influence on ART centers cannot be ignored. Is the influence of the internet on the ART center consistent with the influence of academic standing? What is the gap between them? Whether the internet influences patients' choices, and whether it can attract the attention of the capital market and alter the nature of hospitals in the future have not been examined.

### The Aim of This Study

This study investigated the operation and popularization of the WeChat internet connection by ART centers. We used the technique for order of preference by similarity to ideal solution (TOPSIS) to construct an internet influence measure of ART centers and obtain a Ranking of Internet Influence on Reproductive Centers (RIIRC), which provides guidance for encouraging patients to seek reproductive help at ART centers by enhancing public awareness of them and their public image.

## Methods

### Sample Selection

The initial study sample consisted of 451 ART centers that had been approved by the NHCPRC by the end of 2016. A total of 498 medical institutions were approved by the NHCPRC as of December 31, 2018 [13]. The 47 new medical institutions approved between December 31, 2016, and December 31, 2018, were not included in the study because of their short history.

We began by browsing the official website of each ART center, including its main page and subpages, searching for the WeChat icon, QR codes, and opening announcements. If no WeChat official account was found, we proceeded to the next step, which was to search for sites that contained words or phrases such as “Weixin” or “WeChat.” If the website of the ART center did not have a “search” function, we used Google or Baidu to search within the medical institution’s website.

We also searched directly in the WeChat App using the keywords “name of medical institution,” “reproductive medicine,” “ART center,” or “reproductive medicine department.” Finally, we consulted with the person in charge by emails, messages on the website of ART center, etc. If we confirmed that WeChat account have been applied, they will not be consulted.

These procedures revealed that 200 of the 451 ART centers had opened WeChat official accounts, as of December 31, 2018. Afterwards, Webpage parsing techniques and other technical means were used to obtain basic information for the 200 centers.

The WeChat accounts of the 200 ART centers were screened again, and unqualified accounts were excluded. We examined the text data of the official account posts to see if they met the first exclusion criterion: that is, having multiple values (five or more values) on the 19 indicators described in the section on Index of Internet Influence. A total of 41 accounts that had multiple missing values were excluded. The second criterion was that there was no record value; that is, the account had not been continuously maintained and updated. We found that nine accounts had not pushed tweets or only had a few tweets in the past year, so they were excluded. This left a total of 150 official accounts included in the analysis. All NULL values in the text of tweets of the 150 accounts were replaced with zero for the convenience of subsequent processing.

### Collection of Basic Data

The basic data of the 150 centers were collected manually to evaluate the influence of the internet through the WeChat accounts. We hired 4 assistant researchers to inspect and collect the data after they received professional training.

The account data from each center were randomly divided into four equal parts, and the 4 assistant researchers randomly selected one of them to inspect and collect the data. After one round of collection, they exchanged the list for a second round, and finally inspected the two rounds of data. If a discrepancy was found, it was discussed and consensus was reached.

Basic information included an assessment index for the access to technology, an official account menu bar score, geographical location, date of formation on the department, date of approval by the NHCPRC, date of creating the WeChat official account, and whether there was an official website and official hospital certification (service account and subscription account).

The assessment index for access to technology refers to the type of ART approved by the NHCPRC (Table 1).The official account menu bar score refers to the functions contained in the menu bar of the official account, which generally included a department introduction, appointment registration, navigation, medical examination reports, popular scientific articles, etc. One point was assigned for each function and the sum of the points was the score.

**Table 1:** Types and scores of assisted reproduction technology in medical institutions.

Access to technology	Score
Artificial insemination with husband (AIH)	0.5
Artificial insemination by donor (AID)	0.5
In vitro fertilization and embryo transfer (IVF-VT)	2
Intracytoplasmic sperm injection (ICSI)	3
Preimplantation genetic diagnosis (PGD)	4

### Acquisition, Conversion, and Cleaning of Data

Data mining of WeChat accounts generally requires using appropriate methods and tools, according to the characteristics of the dataset. This study mainly adopted the Webpage parsing technique, using Python 3.7 as the development language, PyChram as the development platform, and MongoDB for data storage. The data had to be further processed and structured for later analysis and mining, mainly involving data deduplication, integration, and transformation.

The data from the eligible centers were from November 1, 2018 to October 31, 2019.

### Index of Internet Influence

In light of the existing research literature and the purpose of this study [16-18], we developed an index to measure the internet influence for ART centers through their WeChat accounts. The 19 indices shown in Table 2 were selected for use in the study.

It was necessary to clarify the concepts and definitions of indices selected in this study. WeChat service account and subscription account can setup to eight tweets at a time. The heading of the main title was called the “headline,” and the seven subtitles were collectively referred to as “subheadlines.” “Posting frequency” (*x*_1_, number of posts per month) equals to the number of articles WeChat account posts in the past year divided by 12. “Number of articles of each post” (*x*_5_, number of articles per post) equals to the total number of articles WeChat accounts posts divided by the number of posts in the past year. “Average number of views of the headlines” (*x*_6_, number of views per article) equals to the total number of views of headline articles divided by the number of headlines in the past year. “Average number of likes of headlines” (*x*_7_, number of likes per article) equals to the total number of likes of the headline articles divided by the number of headlines in the past year. The number of views and likes of posts of the other indicators used a calculation method similar to that of the “Average number of views of the headlines” and “Average number of likes of headlines.”

**Table 2:** The index of internet influence for assisted reproductive technology centers.

Category and index		Symbol	
**Basic index**			
	Posting Frequency		*x*_1_
	Number of articles of each post		*x*_5_
	Menu bar score		*x*_18_
**Headline index**			
	Average number of views of headlines		*x*_6_
	Average number of likes of headlines		*x*_7_
	Average number of views of original headlines		*x*_8_
	Average number of likes of original headlines		*x*_9_
	Average number of views of unoriginal headlines		*x*_10_
	Average number of likes of unoriginal headlines		*x*_11_
**Academic index**			
	Assessment Index for Access Technology		*x*_19_
**Articles type**			
	Number of headlines		*x*_2_
	Number of subheadlines		*x*_3_
	Number with original content		*x*_4_
**Subheadline index**			
	Average number of views of subheadlines		*x*_12_
	Average number of likes of subheadlines		*x*_13_
**Original content**			
	Average number of views of original content		*x*_14_
	Average number of likes of original content		*x*_15_
	Average number of views of unoriginal content		*x*_16_
	Average number of likes of unoriginal content		*x*_17_

### Construction of the Internet Influence Measure

The construction of the measure of internet influence and the output of the figures were based on R Programming Language (The R Foundation Conference Committee, version 3.6.1, 2019-07-05).

The determination of the weight of the index and measurement model were based on TOPSIS.

First, we created an index matrix; there were *n* evaluation objects, and each evaluation object had *m* evaluation indices. It is usually necessary to convert low-quality indices with lower values and better evaluation results to high-quality indices to make the objectives have the same trend. In this study, however, all of the indices were high-quality indices, so conversion did not have to be performed.

**Figure 1:**
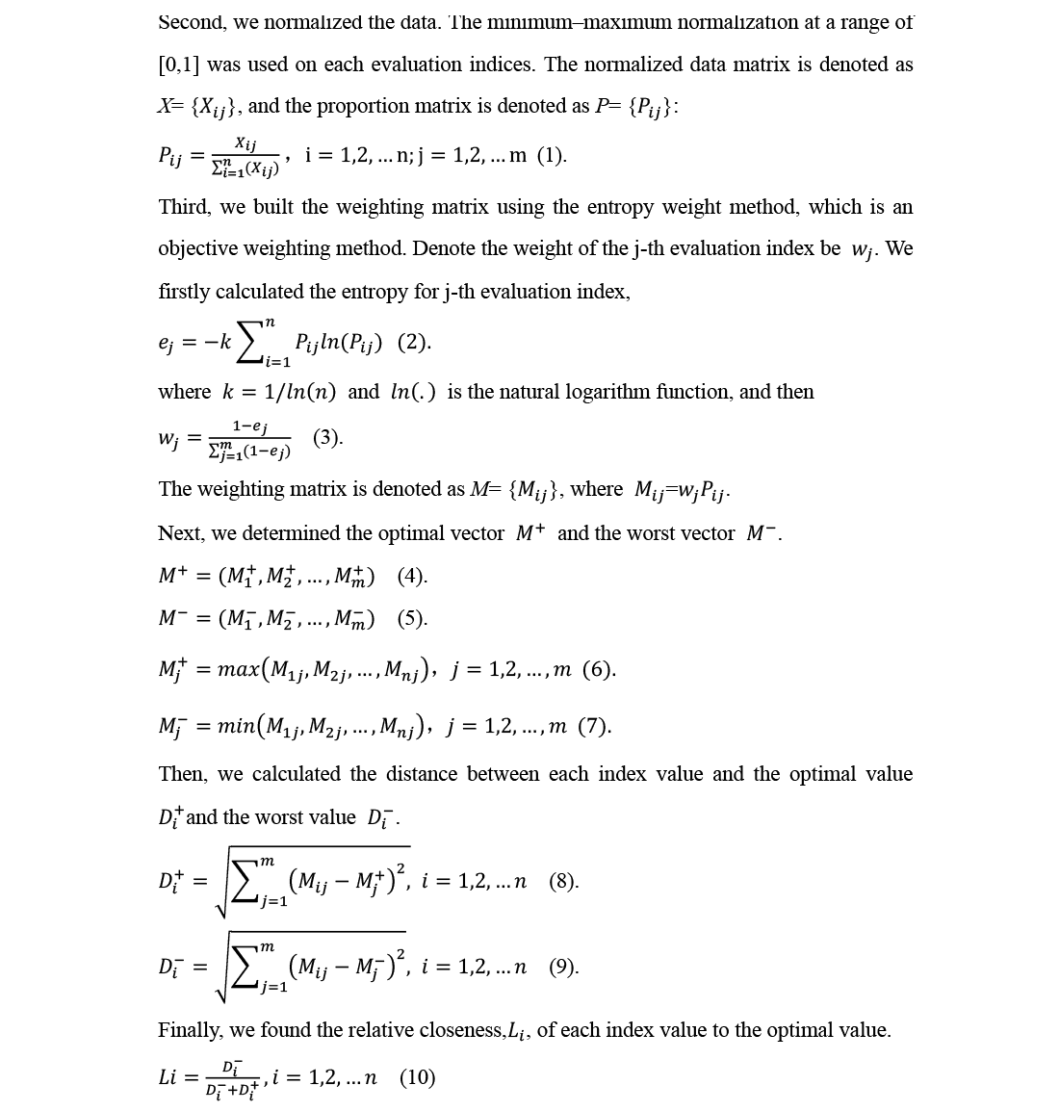
Mathematical equations.

The proportion matrix *P_ij_*, weighting matrix *M_ij_,* and relative closeness *Li* were computed by equations (1), (4), (5), (6), (7), and (10) as shown in Figure 1.

## Results

### Distribution and Technical Ability of the Centers Is Uneven

**Figure 2:**
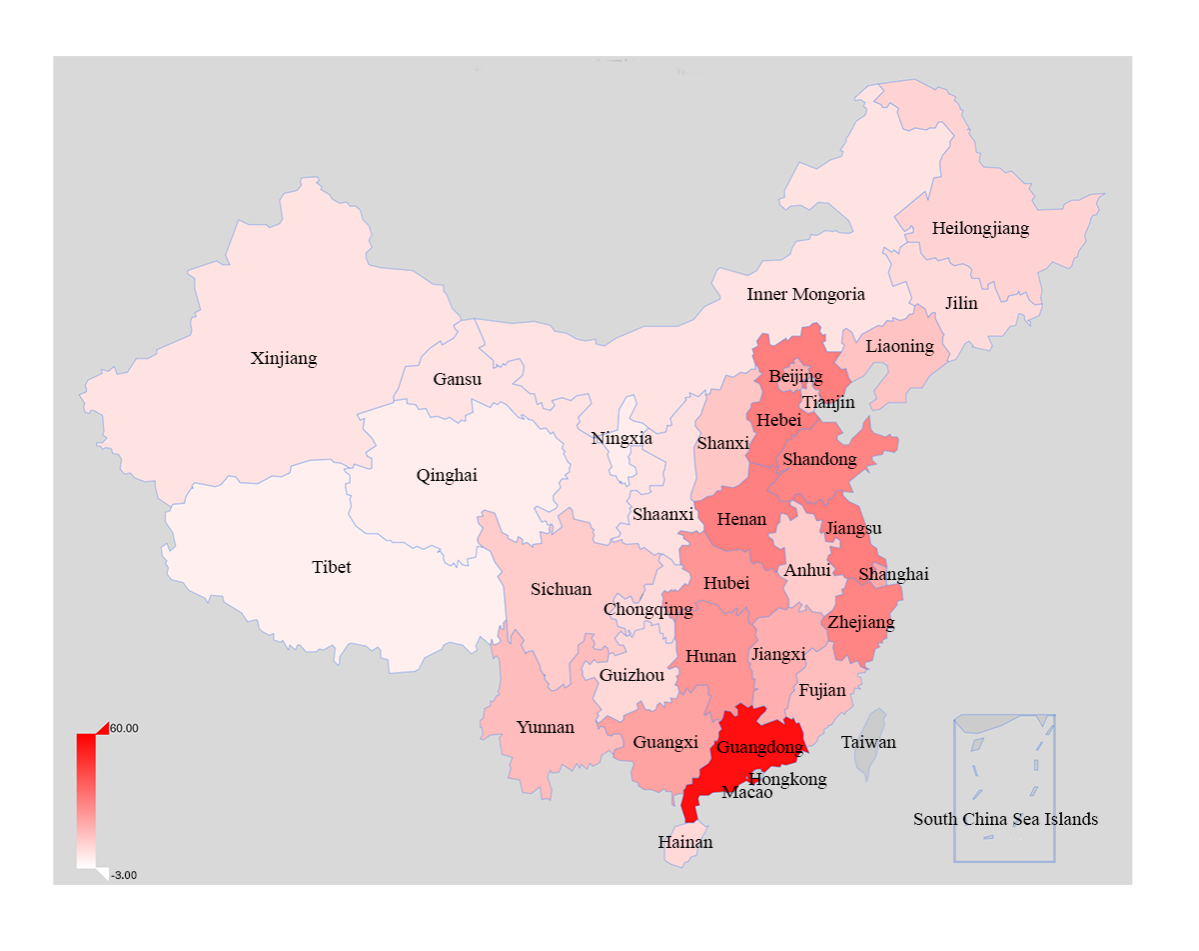
Distribution of 451 assisted reproduction technology center across Mainland China.

The 451 ART centers were located in 31 provincial administrative regions of China, including 22 provinces, five autonomous regions, and four municipalities directly under the central government. However, they were mainly in the southeast coast, the middle and lower reaches of the Yangtze River, and the Bohai Rim (Figure 2).

Nearly three-quarters (327/451, 72.5%) of the ART centers in Chinese performed in vitro fertilization-embryo transfer and intracytoplasmic sperm injection, whereas only 8.9% (40/451) could perform preimplantation genetic diagnosis (Table 3).

The Third Hospital of Beijing Medical University (now known as Peking University Third Hospital) and the Second Clinical Medical College of Hunan Medical University (now known as Xiangya Hospital Central South University) were the earliest medical institutions to conduct ART in China. The number of ART centers increased dramatically from 2007 to 2016, during which time a total of 403 ART centers were approved (Table 4).

**Table 3:** The number of accessible technologies (total is the sum of all access technology).

Access to technology	Value, n (%)
Artificial insemination with husband (AIH)	449 (37.02)
Artificial insemination by donor (AID)	60 (5.00)
In vitro fertilization and embryo transfer (IVF-VT)	327 (26.96)
Intracytoplasmic sperm injection (ICSI)	327 (26.96)
Preimplantation genetic diagnosis (PGD)	40 (3.30)

**Table 4:** The number of ART centers approved by National Health Commission of the People’s Republic of China.

Years	Number, n
2002	12
2004	37
2006	64
2007	95
2011	178
2012	356
2016	451
2018	489

### WeChat Accounts Are Uncommon and Their Operation Is Inadequate

**Figure 3:**
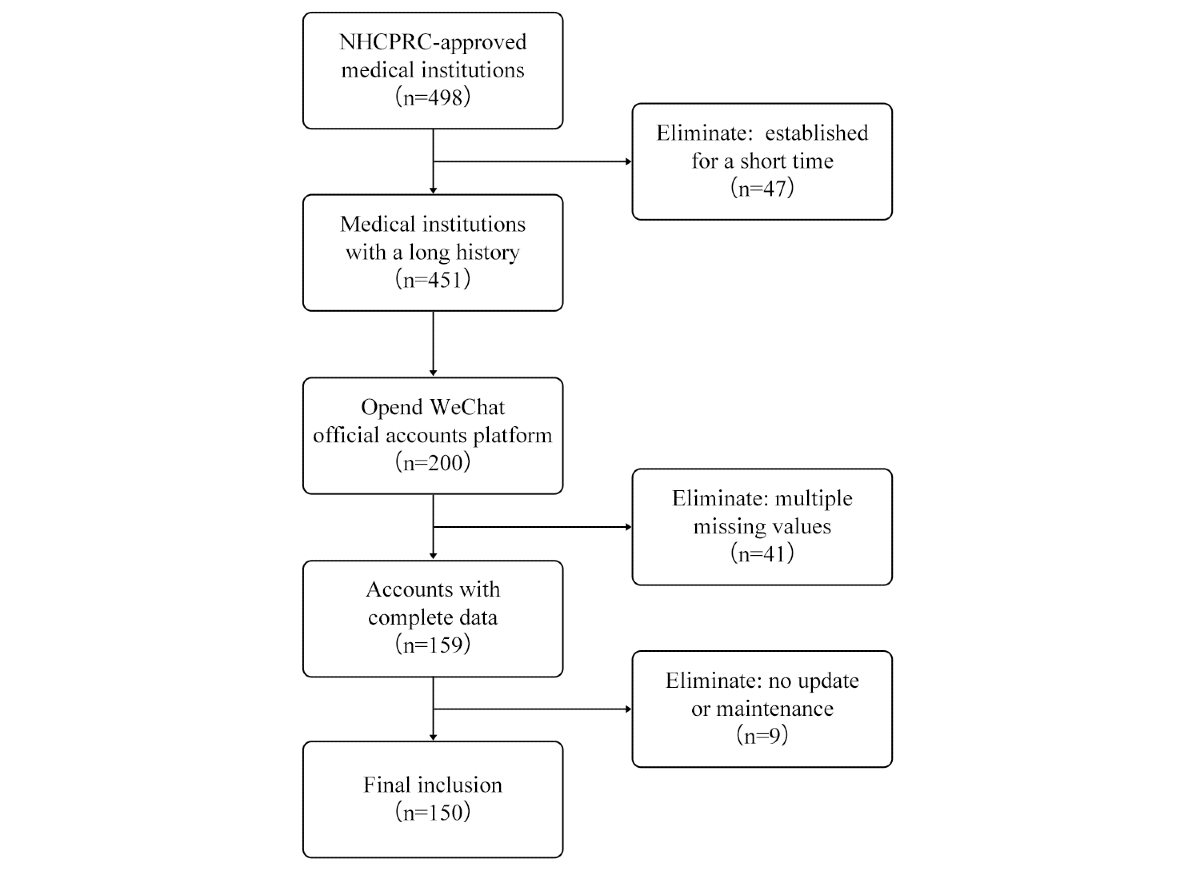
Flowchart of the selection process for WeChat official accounts of assisted reproduction technology centers. NHCPRC: National Health Commission of the People’s Republic of China.

We found only 200 ART centers had opened WeChat official accounts, and the operation of these media accounts differed. Of these 200 accounts, 41 had multiple missing values related to their posts, and another nine accounts had not been continuously maintained, updated, or pushed, or only had a few tweets in the past year. Therefore, only 150 accounts ultimately met the criteria for inclusion in the study (Figure 3).

### Low Usage and Quality of WeChat Accounts

**Figure 4:**
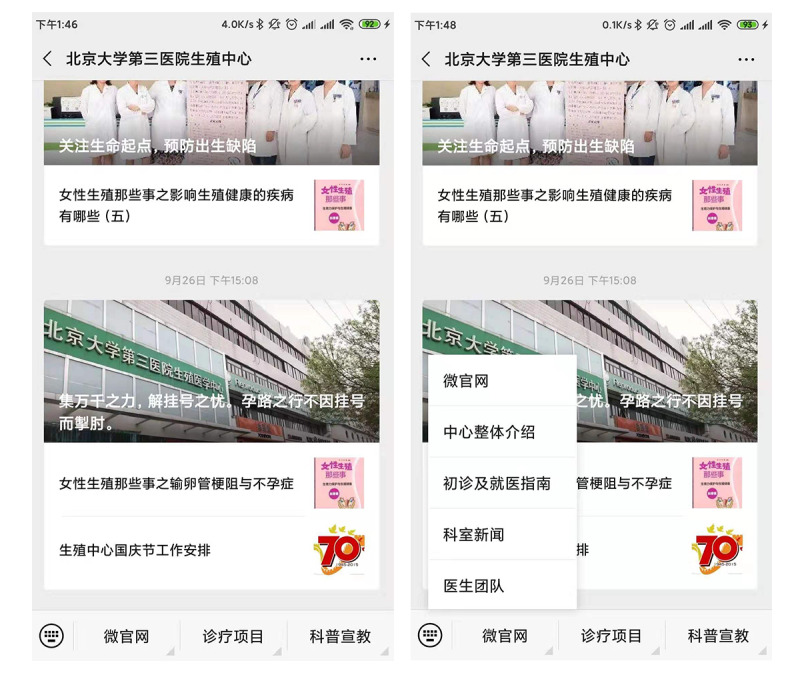
Profile of WeChat official accounts for assisted reproduction technology centers.

The main page of a WeChat official account displays posts, including text, graphics, audio, and video. And there is an area at the bottom is called *menu bar*, through which patients can quickly find what they want (Figure 4). On November 14, 2012, Beijing Baodao Healthcare was the first ART institution to open a WeChat official account. However, the emergence of centers using WeChat official accounts did not attract people’s attention, according to our measures (Table 5). The index of the functionality of the WeChat account menu bar showed that the menu bar scores of most accounts were between 5 and 10 points (Table 6).The first wave of ART centers began in 2013 and reached its peak in 2014, indicating that the awareness and utilization of WeChat official accounts by ART centers needs to be further improved in China. The official website and certification of the WeChat official account is an important indicator of patients’ trust. As shown in Tables 7 and 8, there were 127 (63.5%) and 166 (83.0%) accounts with an official website and certification, respectively.

**Table 5:** The number of WeChat official accounts foundation date.

Years	Number, n
2012	1
2013	7
2014	50
2015	115
2016	144
2017	183
2018	200

**Table 6:** Menu bar scores of WeChat official accounts.

Menu bar scores	Number, n
0~2	13
3~4	15
5~6	44
7~8	67
9~10	40
11~12	15
13~14	4
15~18	2

**Table 7:** The website proportion of WeChat official accounts.

Type	Value, n (%)
With websites	127 (63.5)
Without websites	73 (36.5)

**Table 8:** The proportion of official hospital certifications.

Type	Value, n (%)
Certified	166 (83.0)
Not certified	34 (17.0)

### Extremely Uneven Quantity of Posted Article

As already mentioned, NHCPRC approved 451 medical institutions to conduct human ART, but less than half (200/451, 44.4%) of the ART centers have opened WeChat official accounts, of which 28.4% (128/451) have a subscription account and 16.0% (72/451) have a service account. The overall number of ART centers without an official account is 55.6% (251/451; Multimedia Appendix 1).

In roughly the past year, the cumulative number of posts of these official accounts varied greatly. In ascending order, the medical institutions below the third quartile (Q3) had not posted more than 100 times (Multimedia Appendix 1). Similarly, posting frequency and the number of articles of each post showed the same trend; the Q3 was 5.7 posts per month and 2.9 articles per post (Multimedia Appendix 1).

Afterward, we focused on the number of views of posts. In general, the average number of views of the headlines of the 150 official accounts was 1344.20, whereas the average number of views of the subheadline was only 5.20.

Further analysis of these accounts found that the greatest number of views of the headlines of accounts falling into Q1, Q2, Q3, and Q4 were 375.4, 795.3, 1791.2, and 18445.0, respectively, and the number of views of the subheadline was 128.6, 323.8, 710.0, and 9421.5, respectively (Multimedia Appendix 1). The number of likes was another evaluation index, which measures readers’ recognition and approval of tweets. The number of likes for the headline and subheadlines showed trends similar to the number of views (Multimedia Appendix 1).

### Ranking of Internet Influence

As mentioned earlier, TOPSIS was used to evaluate the internet’s influence for ART centers through their WeChat accounts. To do this, we adopted the objective method to determine the weight of the index (Table 9).

Multimedia Appendix 2 shows the results of the TOPSIS analysis. Among the top 10 medical institutions in the RIIRC are the Reproductive and Genetic Hospital of CITIC-XIANGYA, the Guangdong Institute of Family Planning Science and Technology, The First Affiliated Hospital of Anhui Medical University, the Jiangsu Province Hospital Affiliated with Nanjing Medical University of Medicine, the Peking University Third Hospital, the Kunming IVF Hospital, the Third Affiliated Hospital of Guangzhou Medical University, the Sun Yat-Sen Memorial Hospital of Sun Yat-Sen University, the Northwest Women’ and Children’s Hospital, and the Reproductive Hospital Affiliated with Shandong University.

**Table 9:** The index weight of WeChat official accounts for assisted reproduction technology centers.

Index	Weight
*x*_1_	0.029225
*x*_2_	0.027547
*x*_3_	0.067134
*x*_4_	0.090053
*x*_5_	0.027628
*x*_6_	0.042456
*x*_7_	0.038251
*x*_8_	0.06768
*x*_9_	0.058521
*x*_10_	0.042434
*x*_11_	0.039738
*x*_12_	0.047673
*x*_13_	0.073306
*x*_14_	0.115235
*x*_15_	0.099312
*x*_16_	0.045186
*x*_17_	0.073518
*x*_18_	0.008801
*x*_19_	0.006303

## Discussion

### Principal Findings

Taking WeChat official accounts as the breakthrough point, this study analyzed the information about ART centers from the perspective of a semiprofessional patients, using basic information and the number of views and likes to construct a mathematical model and obtained the RIIRC. The major findings involve the geographic distribution of the ART centers, their technical ability, their use of WeChat, and the internet influence for the centers.

Although the 451 ART centers in the sample are distributed throughout the country, their geographic distribution is uneven, and they vary in terms of types of ART they provide. Analysis of the text of posts in their official accounts found the ART centers have insufficient awareness of network exposure and publicity, and some of the centers’ internet influence rankings are inconsistent with their medical level and academic status. We also found evidence that internet influence may affect the ART market structure in the future and affect the academic rankings of the medical institutions.

### WeChat Official Account Is an Excellent Tool to Popularize Assisted Reproduction Technology Centers

WeChat is an internet instant-messaging social software that was launched by Tencent in January 2011 and launched its official account in July 2012 [19]. As of June 2019, WeChat had 1.08925 billion users who were active every month, and the total number of registered official accounts exceeded 20 million, forming a new type of vast social network, which has increasingly attracted the attention of researchers [20]. In the first half of 2019, the average daily use time of WeChat was 89.8 min, which is 5.1% higher than it was in 2018, making it become one of the apps with the longest daily use time per capita in China [21]. In addition, WeChat has a function that other social software does not have. It is called “Moments (Pengyouquan),” which refers to a circle formed by friends who know each other through WeChat; it is a niche and private circle constructed by an acquaintance relationship chain. This attribute of the “Moments” makes information posted by individuals more likely to be followed and trusted by friends. Given the characteristics of WeChat and WeChat official accounts, we have reasons to believe that WeChat platforms can provide ART centers with a convenient and low-cost opportunity to attract local customers and increase exposure.

### The Selected Indices Are Reasonable to Reflect Ranking of Internet Influence on Reproductive Centers

We chose 19 indices as model parameters based on the framework of the WeChat official account, used an objective method to determine the weight of each index, and used TOPSIS to construct a measurement model of internet influence for ART centers (the RIIRC).Some WeChat official account rankings have recently appeared in China, including the WeChat Communication Index and the New Rank Index [16-18]. These rankings and evaluation methods are based on data available from WeChat official accounts, which reasonably reflect the comprehensive internet influence of official accounts to a certain extent and are generally consistent with our methods.

Although the focus of our research was the WeChat official account for ART centers, we also included an index of access to technology. The weight of the access to technology index in our study was 0.006303, which is very low. The reason is that this index assesses the medical level and academic ability of the ART center; it is not a conventional index of internet influence.

The number of views and likes have been used in capacity research as indices to measure the internet influence. We found that the weights for the number of articles of each post (*x*_5_) and posting frequency (*x*_1_) were 0.027628 and 0.029225, respectively, accounting for a relatively low proportion of influence. There was no obvious correlation between these two indices and propagation, indicating that they affect a small portion of the internet influence on the WeChat official accounts, which is consistent with previous results [22].One possible reason is that posting articles too frequently does not increase the average number of views, nor enhance a good reputation. Indeed, frequent posting may even distract patients’ attention. The majority of the content posted by the WeChat official accounts of the ART centers was popular scientific articles. The number of views of such articles was acceptable, but the number of likes was not high. Psychological research shows that likes are a way for the public to express their approval of content and ideas [23]. The possible reasons why such articles fail to motivate patients’ likes include their scientific nature, the preciseness of posts, and the fitness of the health hotspots. Among the many indices, originality was one of the most important ones for measuring the quality of official accounts and the authors’ writing level, and its weight was relatively high (30% of the total weight). Some studies have shown that the average number of views of original articles is 6.54 times greater that of nonoriginal [24], which is consistent with our results. In addition, the data used in our analyses were the average number of views and likes of posts of official accounts during the past year, which avoids the problem of variations in the number of views and likes over shorter time periods. Moreover, the selected indices are data that can be viewed directly by general patients. Assessing the internet behavior of hospitals from the perspective of users can better and more objectively reflect the internet influence of WeChat official accounts for ART centers. In summary, no matter what type, quantity, or weight of an index is used, it can represent the internet influence of the official account for the ART center to a certain extent.

### Most Assisted Reproduction Technology Centers’ Ranking of Internet Influence on Reproductive Centers Are Inconsistent With Their Medical Level and Academic Status

WeChat launched its official accounts in July 2012, but this tool has not received the attention of ART institutions. By the end of 2018, only 200 ART centers had opened official accounts, and only 150 had operated and maintained them normally. The RIIRC showed that the Reproductive and Genetic Hospital of CITIC-XIANGYA is at the top of the list with 100 points. The Guangdong Institute of Family Planning Science and Technology (66.08), the First Affiliated Hospital of Anhui Medical University (65.86), the Jiangsu Province Hospital Affiliated with Nanjing Medical University of Medicine (50.22), and the Peking University Third Hospital (46.86) are among the top five. At present, the most authoritative list of medical institutions in China is the “Fudan University Hospital Ranking,” released in November 2019, which includes rankings of the reproductive medicine specialty [25]. Those rankings focus on discipline structure, clinical technique and medical quality, and the level of scientific research of each hospital. The ART hospitals at the top of these two lists are mainly distributed in the coastal areas of Southeast China and the Bohai Rim region. The ART centers in these areas are rated significantly higher than those of other regions in terms of their opening proportion and internet influence. This may be because of the superior resources, such as open policies, sufficient techniques, and financial support [26,27].

### Ranking of Internet Influence on Reproductive Centers May Indicate the Future Market Position of Assisted Reproduction Technology Centers

The Peking University Third Hospital and the Reproductive and Genetic Hospital of CITIC-XIANGYA were the earliest medical institutions to provide ART in China. The first two test-tube babies in mainland China were born in 1988, and these two hospitals were the domestic leaders of ART [28,29]. The ART center of the Peking University Third Hospital has published more than 100 scientific articles in internationally renowned magazines, such as *Nature*, *The Lancet*, *Cell*, and *Proceedings of the National Academy of Sciences*, and it is the most powerful ART center in China [30-33]. The ART cycle of the Reproductive and Genetic Hospital of CITIC-XIANGYA reached 49,000 cases in 2018. In the first quarter of 2019, this increased by 14.84% compared with the same period of last year, making it ART center with the most comprehensive medical technology and the largest number of patients at home and abroad [29]. The Peking University Third Hospital and Reproductive and Genetic Hospital of CITIC-XIANGYA are both at the forefront of the two rankings. They not only lead in terms of scientific research and clinical technique but also attach great importance to internet influence. We have reason to believe that such ART centers can maintain and further enhance their level of medical care, academic status, and internet reputation, so as to expand market advantages and become leaders in ART in China.

The Guangdong Institute of Family Planning Science and Technology, the Third Affiliated Hospital of Guangzhou Medical University, and Sun Yat-Sen Memorial Hospital Sun Yat-Sen University are all located in Guangdong Province of China, ranking 2nd, 7th, and 8th in RIIRC, respectively. The Third Affiliated Hospital of Guangzhou Medical University was ranked 20th in the Fudan University Hospital Ranking, and the other two hospitals were not nominated. There are two possible reasons for the above phenomenon: (1) Guangdong, as the province with the largest gross domestic product in China, has a developed economy and a strong business sense, and it recognizes the importance of public praise and (2) Guangdong Province has a large number of ART centers (as many as 56), and the intensively competitive situation made the ART centers aware of the importance of survival, so they paid attention to their image and reputation on the internet. Jiangsu Province Hospital Affiliated with Nanjing Medical University and The First Affiliated Hospital of Anhui Medical University are ranked 4th and 3rd in RIIRC, respectively, while they rank 9th and 13th in the Fudan University Hospital Rankings, respectively. Both of these ART centers have been established for a long time, have a high level of medical care, and a strong sense of subjective development. With the continuous increase of internet exposure opportunities, the number of patients will definitely increase, which will also improve the clinical technique and medical quality of ART centers. We have reason to believe that the aforementioned ART centers will continue to be important players and the front runners in the ART market in the region by focusing on academic development and online exposure.

We also found another interesting phenomenon, which is that some hospitals are at the top of Fudan University Hospital rankings but in the middle and lower range of the RIIRC. These ART centers are at the forefront in terms of clinical technique and scientific research, but their WeChat official accounts are not well operated, and the number of views is lower. For some public hospitals, though there are still many patients who can help them maintain their market position in the region, the long-term adoption of this approach will lead to the loss of patients, which will lead to their annexation by other public medical institutions or private hospitals

Although the NHCPRC has approved 498 ART centers, the existing medical institutions are far from meeting the needs of the market, and private hospitals and private capital have targeted this market.

Listed company Jinxin Fertility and internet medical platform WeDoctor conducted ART technology services to provide patients with high-quality and personalized treatment programs; its Chengdu Xinan Gynecology Hospital and Kunming IVF Hospital are among the top 10 in China. These private hospitals joined the top 10 in a short period of time; one important reason is that they pay attention to network publicity. Once they have more technology, talent, and capital, they are likely to become leaders in the field of ART, and even surpass the top public medical institutions in the region, because the general public does not know much about the academic standing.

### Summary and Limitation

In addition, this study found that in the media era, WeChat can provide hospitals with a convenient and low-cost opportunity to attract customers, as well as provide an easy and efficient way to promote their brand and market. Generally speaking, the following three problems exist.

First, the earliest ART centers using WeChat official accounts were newly established hospitals, rather than older ones, probably because the new hospitals were more open to new technologies. Medical institutions with a long history prefer to focus on patient safety, such as reducing medical malpractice, rather than increasing opportunities to communicate with patients.

Second, in the information age, users have more opportunities to obtain various types of information and less patience for reading. For the WeChat account of an ART center, it is necessary to grasp the frequency, time and user needs of the post, and cultivate the user’s website “stickiness” and reading habits through a fixed post mode.

Third, opening and verifying an official account of an ART center on the WeChat app is only the first step for a medical institution to take action. In fact, some hospitals stop updating their accounts after only a few tweets. Although some scholars have proposed detailed guidelines for the use of social media in medical institutions and answering patients’ difficult questions, most ART centers do not follow these recommendations [34,35].

The sample size of the ART centers included in this study’s sample was limited by two factors. First, although the NHCPRC had approved 498 ART centers by July 4, 2019, 47 new medical institutions were not included in the study because they only had a short history. Other centers had to be excluded because they had incomplete data. Future research should include more ART centers and compare their use of WeChat official accounts and changes over time. Second, all the information used in this study was based on public data, and the cycle number of each ART center has not been reported. The reason for this is that the reporting system of the Chinese Society of reproductive medicine is for internal use only. In addition, the WeChat official accounts of some ART centers were registered in the names of individuals rather than the hospitals or departments, so these official accounts did not fall within the scope of this study.

Although this study has some limitations, we analyzed and compared the operation of WeChat official accounts for ART centers and considered the combined academic influence, which offers guidance for promoting patients’ access to these hospitals by strengthening their public image and exposure and a reference point for patients to choose suitable ART medical services. It also analyzed the market situation and future competitive advantage of domestic ART centers and indicated ways in which potential investment institutions can enter the field.

AcknowledgmentsThis work was supported by grants from the National Natural Science Foundation (81774075) of China and Priority Academic Program Development of Jiangsu Higher Education Institutions.

## Appendix

Multimedia Appendix 1 The number of views and likes of posts of WeChat official accounts. (a) The proportion of WeChat official accounts. (b) Total times of posts. (c) Posting frequency. (d) The number of articles of each post. (e) Average number of views of headlines. (f) Average number of likes of headlines. (g) Average number of views of subheadlines. (h) Average number of likes of subheadlines.

Multimedia Appendix 2 The ranking of internet influence for assisted reproduction technology centers based on technique for order of preference by similarity to ideal solution.

## Abbreviations

ART: assisted reproduction technology
IVF: in vitro fertilization
NHCPRC: National Health Commission of the People’s Republic of China
RIIRC: Ranking of Internet Influence on Reproductive Centers
TOPSIS: Technique for Order of Preference by Similarity to Ideal Solution

## References

[1] Junqing W, Qiuying Y, Jianguo T, Wei Y, Liwei B, Yuxian L, Yumei Z, Kangshou Y, Weiqun L, Lu C, Ersheng G (2002). Reference value of semen quality in Chinese young men. Contraception.

[2] Liu J, Larsen U, Wyshak G (2005). Prevalence of primary infertility in China: in-depth analysis of infertility differentials in three minority province/autonomous regions. J Biosoc Sci.

[3] Duron S, Slama R, Ducot B, Bohet A, Sørensen DN, Keiding N, Moreau C, Bouyer J (2013). Cumulative incidence rate of medical consultation for fecundity problems--analysis of a prevalent cohort using competing risks. Hum Reprod.

[4] Schmidt L, Sobotka T, Bentzen JG, Andersen AN, ESHRE ReproductionSociety Task Force (2012). Demographic and medical consequences of the postponement of parenthood. Hum Reprod Update.

[5] Boivin J, Bunting L, Collins JA, Nygren KG (2007). International estimates of infertility prevalence and treatment-seeking: potential need and demand for infertility medical care. Hum Reprod.

[6] Bhattacharya S, Porter M, Amalraj E, Templeton A, Hamilton M, Lee AJ, Kurinczuk JJ (2009). The epidemiology of infertility in the North East of Scotland. Hum Reprod.

[7] Slade P, O'Neill C, Simpson AJ, Lashen H (2007). The relationship between perceived stigma, disclosure patterns, support and distress in new attendees at an infertility clinic. Hum Reprod.

[8] Bak CW, Seok HH, Song SH, Kim ES, Her YS, Yoon TK (2012). Hormonal imbalances and psychological scars left behind in infertile men. J Androl.

[9] Merritt MA, de Pari M, Vitonis AF, Titus LJ, Cramer DW, Terry KL (2013). Reproductive characteristics in relation to ovarian cancer risk by histologic pathways. Hum Reprod.

[10] Jensen TK, Jacobsen R, Christensen K, Nielsen NC, Bostofte E (2009). Good semen quality and life expectancy: a cohort study of 43,277 men. Am J Epidemiol.

[11] Wu AK, Elliott P, Katz PP, Smith JF (2013). Time costs of fertility care: the hidden hardship of building a family. Fertil Steril.

[12] State Council (2007). China government_central government portal.

[13] (2019). National Health Commission of the People's Republic of China.

[14] (2015). National Health Commission of the People's Republic of China.

[15] CNNIC (2019). China Internet Network Information Center.

[16] Qingping L [GSDATA - Qingbo Big Data].

[17] Xu N (2019). Newrank.

[18] Yueming Y, Pengwei Z (2016). A Method for Evaluating the Influence of the WeChat Public NO. JOURNAL OF INTELLIGENCE.

[19] [WeChat is a Way of Life].

[20] (2019). WeChat, CAICT, DCRC.

[21] (2018). QuestMobile.

[22] Jing F, Wei L (2016). A Study on Influential Factors of Wechat Public Accounts Information Transmission Hotness. JOURNAL OF INTELLIGENCE.

[23] Jing L (2016). [Emotions and cultural expressions behind WeChat likes]. New Media Res.

[24] Ruoxi Z, Xiaoyan M, Ning L, Lili G, Songlin W, Liang C, Weina Y, Wenjun M (2018). Analysis on released messages and its effect on WeChat accounts of public health category. Chinese Journal of health Education.

[25] (2018). Fudan University.

[26] Ma L, Xie Q, Shi S, Ye X, Zhao A (2017). Regional maldistribution of China’s hospitals based on their structural system. Sustainability.

[27] Zhang T, Xu Y, Ren J, Sun L, Liu C (2017). Inequality in the distribution of health resources and health services in China: hospitals versus primary care institutions. Int J Equity Health.

[28] Peking University Third Hospital.

[29] CITIC Xiangya Reproductive and Genetic Specialty Hospital.

[30] Guo F, Yan L, Guo H, Li L, Hu B, Zhao Y, Yong J, Hu Y, Wang X, Wei Y, Wang W, Li R, Yan J, Zhi X, Zhang Y, Jin H, Zhang W, Hou Y, Zhu P, Li J, Zhang L, Liu S, Ren Y, Zhu X, Wen L, Gao YQ, Tang F, Qiao J (2015). The transcriptome and DNA methylome landscapes of human primordial germ cells. Cell.

[31] Guo H, Zhu P, Yan L, Li R, Hu B, Lian Y, Yan J, Ren X, Lin S, Li J, Jin X, Shi X, Liu P, Wang X, Wang W, Wei Y, Li X, Guo F, Wu X, Fan X, Yong J, Wen L, Xie SX, Tang F, Qiao J (2014). The DNA methylation landscape of human early embryos. Nature.

[32] Hou Y, Fan W, Yan L, Li R, Lian Y, Huang J, Li J, Xu L, Tang F, Xie X, Qiao J (2013). Genome analyses of single human oocytes. Cell.

[33] Qiao J, Li R (2014). Fertility preservation: challenges and opportunities. Lancet.

[34] Bertot JC, Jaeger PT, Hansen D (2012). The impact of polices on government social media usage: Issues, challenges, and recommendations. Gov Inf Q.

[35] Effing R, Spil TA (2016). The social strategy cone: Towards a framework for evaluating social media strategies. Int J Inf Manag.

