# Supplementary figures and images for: Internet Influence of Assisted Reproduction Technology Centers in China: Qualitative Study Based on WeChat Official Accounts
Source: J Med Internet Res. 2020 Jun 10;22(6):e17997. doi: 10.2196/17997 (PMC7315359; doi:10.2196/17997)

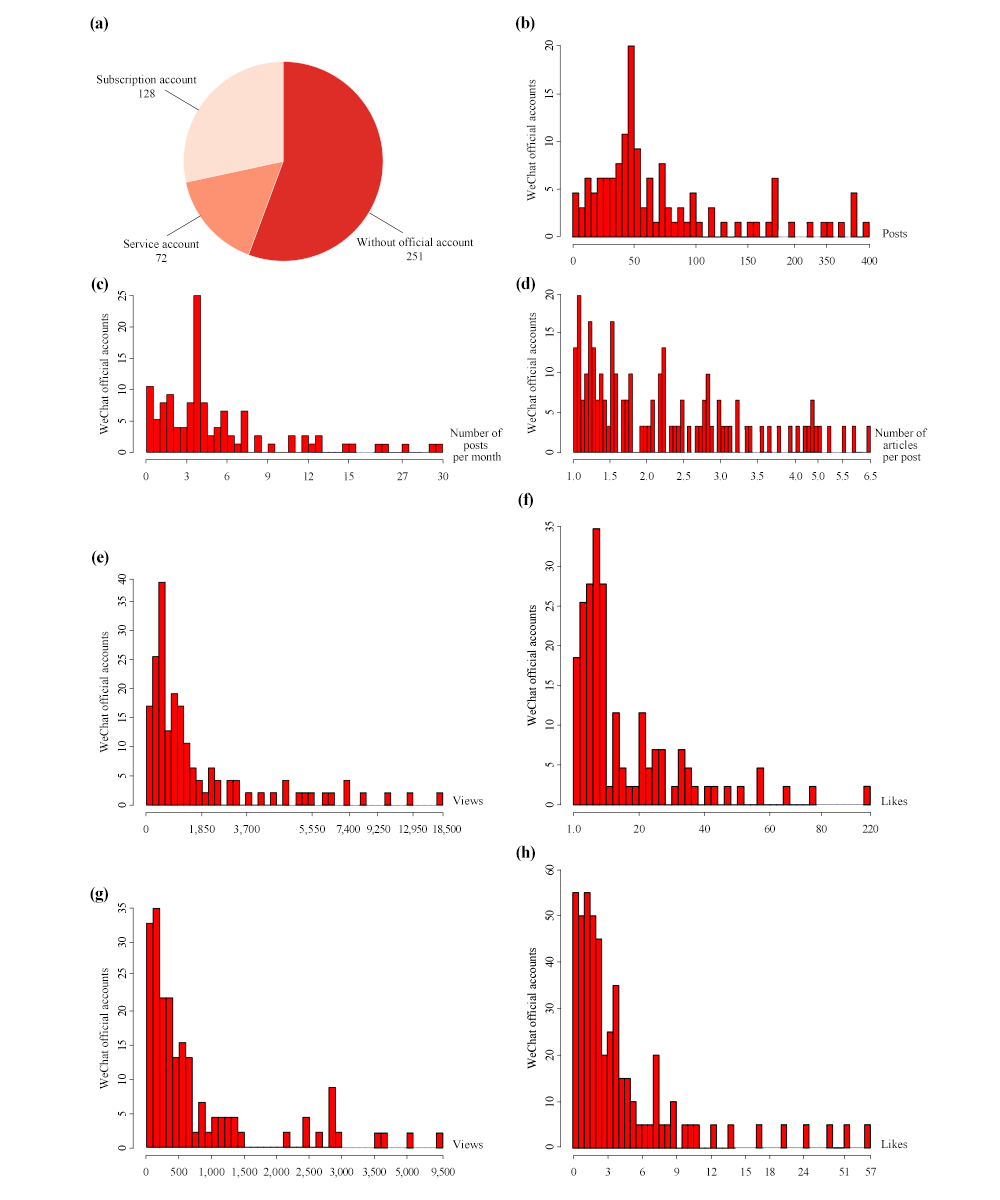

Supplement: Multimedia Appendix 1 [file jmir_v22i6e17997_app1.png]
