# Supplementary material for: Internet Influence of Assisted Reproduction Technology Centers in China: Qualitative Study Based on WeChat Official Accounts
Source: J Med Internet Res. 2020 Jun 10;22(6):e17997. doi: 10.2196/17997 (PMC7315359; doi:10.2196/17997)
Supplement: Multimedia Appendix 2 [file jmir_v22i6e17997_app2.docx]

**Multimedia appendix 2.** The ranking of Internet influence for assisted reproduction technology centers based on technique for order of preference by similarity to ideal solution.

| Assisted reproduction technology center | Scores | Ranking |
| --- | --- | --- |
| Reproductive and Genetic Hospital of CITIC-XIANGYA | 100.00 | 1 |
| Guangdong Institute of Family Planning Science and Technology | 66.08 | 2 |
| The First Affiliated Hospital of Anhui Medical University | 65.86 | 3 |
| Jiangsu Province Hospital Affiliated with Nanjing Medical University of Medicine | 50.22 | 4 |
| Peking University Third Hospital | 46.86 | 5 |
| Kunming IVF Hospital | 46.48 | 6 |
| The Third Affiliated Hospital of Guangzhou Medical University | 46.26 | 7 |
| Sun Yat-Sen Memorial Hospital Sun Yat-Sen University | 44.06 | 8 |
| Northwest Women’s and Children’s Hospital | 42.84 | 9 |
| Reproductive Hospital Affiliated to Shandong University | 41.47 | 10 |
| Nanjing Gulou Hospital | 40.47 | 11 |
| Chifeng Obstetrics and Gynecology Hospital | 39.85 | 12 |
| The Sixth Afflicted Hospital, SunYat-Sen University | 39.60 | 13 |
| The First Affiliated Hospital of Zhengzhou University | 35.69 | 14 |
| Tianjin Aiwei Hospital | 31.78 | 15 |
| The First Affiliated Hospital, Sun Yat-sen University | 30.18 | 16 |
| Chengdu Xinan Gynecology Hospital | 29.11 | 17 |
| Shiyan Renmin Hospital | 26.79 | 18 |
| Shanghai JIAI Genetics and IVF Institute | 26.22 | 19 |
| Shanghai First Maternity and Infant Hospital | 25.84 | 20 |
| West China Second University Hospital，Sichuan University | 24.92 | 21 |
| Xingtai Infertility Specialized Hospital | 24.34 | 22 |
| Maternal and Child Care of Qinhuangdao | 24.30 | 23 |
| Liuzhou Maternity and Child Healthcare Hospital | 24.28 | 24 |
| Guangdong Maternity and Child Health Care Hospital | 22.20 | 25 |
| Jinjiang Maternity and Child Health Hospital | 21.15 | 26 |
| The 2nd Affiliated Hospital and Yuying Children's Hospital of WMU | 20.68 | 27 |
| The General Hospital of Northern Theater Command PLA | 19.44 | 28 |
| Guangxi Health and Family Planning Commission | 19.35 | 29 |
| Shenzhen Zhongshan Urology Hospital | 18.16 | 30 |
| Haikou Mary Hospital | 17.96 | 31 |
| Center for Reproductive Medicine, Tongji Medical College, HUST | 17.22 | 32 |
| The Second Nanning People’s Hospital | 17.02 | 33 |
| Women's Hospital School of Medicine Zhejiang University | 16.91 | 34 |
| Harbin First Hospital | 15.96 | 35 |
| Shen Zhen Heng Sheng Hospital | 15.09 | 36 |
| Ruijin Hospital | 14.97 | 37 |
| Zhengzhou Longhai Hospital | 14.17 | 38 |
| The General Hospital of Eastern Theater Command PLA | 13.10 | 39 |
| First People’s Hospital of Yunnan Province | 12.86 | 40 |
| Shenyang Women’s and Children’s Hospital | 12.51 | 41 |
| Haidian Maternal and Child Health Hospital | 12.41 | 42 |
| The Third Affiliated Hospital of Zhengzhou University | 12.07 | 43 |
| Tangdu Hospital | 11.66 | 44 |
| Henan Provincial Reproductive Hospital | 11.48 | 45 |
| The Maternal and Child Health Hospital of Guangxi Zhuang Autonomous Region | 11.42 | 46 |
| Hebei maternity hospital | 11.28 | 47 |
| Kaifeng Reproductive Health and Infertility Hospital | 11.01 | 48 |
| Jiangmen Central Hospital Affiliated Jiangmen Hospital of Sun Yat-Sen University | 10.46 | 49 |
| Guangzhou Women and Children’s Medical Center | 10.37 | 50 |
| Kunming City Maternal and Child Health hospital | 9.71 | 51 |
| Southern Medical University Nangfang Hospital | 9.65 | 52 |
| Peking University Shenzhen Hospital | 9.58 | 53 |
| Chengdu Women’s and Children’s Central Hospital | 9.33 | 54 |
| The Second Affiliated Hospital of Zhengzhou University | 9.19 | 55 |
| The First Hospital of Qiqihar City | 9.03 | 56 |
| Zhejiang University School of Medicine Sir Run Run Shaw Hospital | 9.01 | 57 |
| Shandong Maternal and Child Health Care hospital | 8.94 | 58 |
| Beijing Jiaen hospital | 8.90 | 59 |
| Changsha Hospital of Reproductive Medicine | 8.90 | 60 |
| Zhejiang Provincial People’s Hospital | 8.46 | 61 |
| Gansu Provincial Maternity and Child Care Hospital | 8.43 | 62 |
| The First Affiliated Hospital of Soochow University | 8.39 | 63 |
| Jiangxi Maternal and Child Health Hospital | 8.39 | 64 |
| The Second Affiliated Hospital of Fujian Medical University | 8.31 | 65 |
| Suzhou Municipal Hospital | 8.26 | 66 |
| Anhui Provincial Hospital | 8.15 | 67 |
| Taihe Hospital | 7.91 | 68 |
| Calmette Hospital and The First Hospital of Kunming | 7.78 | 69 |
| Shijiazhuang Obstetrics and Gynecology Hospital | 7.69 | 70 |
| Ningbo Women and Children’s Hospital | 7.62 | 71 |
| Yantai Mountain Hospital | 7.51 | 72 |
| The First Hospital of Jilin University | 7.46 | 73 |
| Baoding Maternal and Child Health Hospital | 7.44 | 74 |
| Zhaoqing Xijiang Hospital | 7.39 | 75 |
| Angel Women’s and Children’s Hospital | 7.19 | 76 |
| Cangzhou Hospital of Integrated TCM-WM Hebei | 7.14 | 77 |
| Wanbei Coal-electricity Group General Hospital | 6.93 | 78 |
| Shenyang Jiuzhou Family Hospital | 6.79 | 79 |
| Wuhan Kangjian Women and Infants Hospital | 6.50 | 80 |
| Sichuan Provincial People’s Hospital | 6.27 | 81 |
| Xiangtan Central Hospital | 6.09 | 82 |
| Maternity and Child Health Care of Zaozhuang | 6.08 | 83 |
| Chenzhou NO.1 People’s Hospital | 6.05 | 84 |
| Luoyang Central Hospital | 6.03 | 85 |
| Maternity and Child Health Care of Yulin | 5.97 | 86 |
| Maternity and Child Health Care of Yueyang | 5.91 | 87 |
| Maternity and Child Health Care of Hubei Province | 5.88 | 88 |
| Luoyang Maternal and Child Health Hospital | 5.83 | 89 |
| Xiamen Maternal and Child Health Care Hospital | 5.79 | 90 |
| Yijishan Hospital of Wannan Medical Hospital | 5.64 | 91 |
| Chengfeng Hospital of Daqing Oilfield | 5.59 | 92 |
| The First Hospital of Harbin Medical University | 5.57 | 93 |
| Women and Children’s Health Care Hospital of Linyi | 5.54 | 94 |
| Huizhou Municipal Central Hospital | 5.40 | 95 |
| The First Affiliated Hospital of Hainan Medical University | 5.37 | 96 |
| The Affiliated Hospital (Group) of Putian University | 5.34 | 97 |
| The First People’s Hospital of Yueyang | 5.29 | 98 |
| Ganzhou Maternity and Child Health Hospital | 5.15 | 99 |
| Tianjin Central Hospital of Gynecology Obstetrics | 4.87 | 100 |
| Baodao Healthcare | 4.85 | 101 |
| SUNLOVE Maternity Hospital | 4.70 | 102 |
| Tangshan Maternal and Child Health Hospital | 4.60 | 103 |
| Hebei Reproductive Medicine Center | 4.59 | 104 |
| Shanghai Tenth People’s Hospital | 4.52 | 105 |
| Maoming People’s Hospital | 4.42 | 106 |
| Nanchang Reproductive Hospital | 4.36 | 107 |
| The People’s Hospital of Guangxi Zhuang Autonomous Region | 4.36 | 108 |
| Jiaozuo Maternal and Child Health Care Hospital | 4.28 | 109 |
| Reproductive Hospital of Weifang Medical University | 4.17 | 110 |
| Foshan Women and Children Hospital | 4.12 | 111 |
| The Affiliated Hospital of Inner Mongolia Medical University | 4.11 | 112 |
| First Affiliated Hospital of Kunming Medical University | 4.00 | 113 |
| Xiangyang Central Hospital | 3.80 | 114 |
| The Second People’s Hospital of Liaocheng | 3.67 | 115 |
| The Affiliated Hospital of Qingdao University | 3.64 | 116 |
| The Maternal and Child Health Care Hospital of Xiantan City | 3.49 | 117 |
| The Second Affiliated Hospital of Soochow University | 3.43 | 118 |
| The First Affiliated Hospital. Zhejiang University | 3.42 | 119 |
| EMBO Hospital | 3.28 | 120 |
| Shenzhen Luohu Hospital | 3.13 | 121 |
| The First Hospital of Hebei Medical University | 3.11 | 122 |
| Wenzhou People’s Hospital | 3.10 | 123 |
| Huien Hospital | 3.07 | 124 |
| Hainan Maternal and Children’s Medical Center | 3.06 | 125 |
| Luohe Central Hospital | 2.99 | 126 |
| Handan Maternal and Child Health Hospital | 2.83 | 127 |
| Taizhou People’s Hospital | 2.81 | 128 |
| Anyang Maternal and Child Health Care Hospital | 2.77 | 129 |
| The First People’s Hospital of Foshan | 2.67 | 130 |
| Handan Central Hospital | 2.63 | 131 |
| The Affiliated Hospital of Beihua University | 2.54 | 132 |
| Xuzhou Central Hospital | 2.54 | 133 |
| The University of Hong Kong-Shenzhen Hospital | 2.54 | 134 |
| The Third Affiliated Hospital of Sun Yat-Sen University | 2.44 | 135 |
| Hangzhou Fuyang Women and Children Hospital | 2.43 | 136 |
| Zhuzhou Central Hospital | 2.35 | 137 |
| Shenzhen People’s Hospital | 2.32 | 138 |
| Shenzhen Maternity and Child Healthcare Hospital | 2.30 | 139 |
| Taiyuan Central Hospital | 2.22 | 140 |
| Mianyang Central Hospital | 2.09 | 141 |
| Wuhai Maternity and Child Health Care Hospital | 2.02 | 142 |
| Qingzhou Maternity and Child Healthcare Hospital | 1.97 | 143 |
| Jiaxing Maternity and Child Health Care Hospital | 1.94 | 144 |
| Yichang Central People’s Hospital | 1.86 | 145 |
| Women and Children’s Hospital of AnShan City | 1.71 | 146 |
| Women and Infants Hospital of Zhengzhou | 1.38 | 147 |
| Suizhou Central Hospital | 1.35 | 148 |
| Changchun Obstetrics-Gynecology Hospital | 1.25 | 149 |
| The Sixth Affiliated Hospital of Guangzhou Medical University | 1.15 | 150 |
